# Effects of Electroconvulsive Therapy on Glycemic Control in Type 1 Diabetes

**DOI:** 10.7759/cureus.30222

**Published:** 2022-10-12

**Authors:** Simona Stefan, Yazan Alzedaneen, Hilary B Whitlatch, Rana Malek, Kashif Munir

**Affiliations:** 1 Department of Medicine, Albert Einstein College of Medicine, New York, USA; 2 Department of Medicine, University of Maryland Medical Center Midtown Campus, Baltimore, USA; 3 Endocrinology, Diabetes and Metabolism, University of Maryland School of Medicine, Baltimore, USA

**Keywords:** insulin-dependent diabetes mellitus, glycemic control, transient hyperglycemia, type 1 diabetes mellitus (t1dm), depression and diabetes, electro-convulsive therapy

## Abstract

Electroconvulsive therapy (ECT) is a treatment modality for refractory depression and other severe psychiatric diseases. Depression is a common comorbid condition of diabetes. Yet, evidence regarding the effect of ECT on glycemic control in patients with diabetes is limited and conflicting, with reports of both exacerbation and amelioration of hyperglycemia. A 52-year-old Caucasian man with a history of type 1 diabetes mellitus (T1DM) was admitted for ECT therapy in the setting of worsening depression refractory to medical treatment. Pre-admission glycemic control was poor. He had significant glycemic variability during his hospitalization with hyper- and hypoglycemia. He required near-daily adjustment of insulin doses and distinct “ECT day” and “non-ECT day” insulin regimens. By the conclusion of his ECT course, in addition to achieving favorable psychiatric recovery, he had a marked improvement in glycemic control. This suggests that the treatment of depression may have beneficial effects on improving glycemic control in patients with T1DM.

## Introduction

Depression affects up to one in three people with diabetes mellitus and has a significant negative impact on clinical outcomes and quality of life [1]. Recognition and appropriate treatment of concurrent psychiatric disease could theoretically prevent the development of diabetes, as well as, reduce medical complications and the cost of treatment. While studies have been mixed as to the effect of medical intervention and psychotherapy on glycemic control in patients with diabetes [2-3], data are lacking as to the effect of ECT on glycemic control. Here we describe glycemic trends in a patient with type 1 diabetes mellitus (T1DM), and severe resistant depression treated with ECT. This case was presented as an abstract at the Endocrine Society Meetings on March 18, 2018. 

## Case presentation

A 52-year-old Caucasian man with type 1 diabetes was admitted for inpatient electroconvulsive therapy (ECT) in the setting of worsening depression refractory to medical treatment. The home insulin regimen included glargine 54 units twice a day and aspart 10 units before each meal. He typically ate one meal a day with an unmeasured calorie intake. A total daily insulin dose of 1.4 units of insulin/kg indicates significant insulin resistance. Pre-admission glycemic control was poor with blood sugars in the 200s-300s mg/dL, although intermittent unpredictable hypoglycemia also occurred. Hemoglobin A1c (HbA1c) was not available. Symptoms at admission included fatigue and severe depression with suicidal ideation. 

Medical history was significant for type 1 diabetes since the age of 10 complicated by diabetic retinopathy, hypertension, and depression with anxiety. Medications included insulin, bupropion, carbamazepine, lorazepam, and venlafaxine. He reported occasional marijuana use and no alcohol use. There was a family history of thyroid disease, depression, hypertension, and cancer.

The patient was afebrile with a mildly elevated blood pressure of 148/78mm Hg, heart rate of 86 beats per minute (bpm), and BMI of 23 kg/m^2^ (height 5’4” = 162cm/weight 136lb = 62kg). Appeared older than the stated age, with a flat affect and no engagement in conversation. The rest of the general examination was normal. Complete blood count, electrolytes, liver function tests, and kidney function were within normal limits as per age, gender, and racial background.

**Figure 1:**
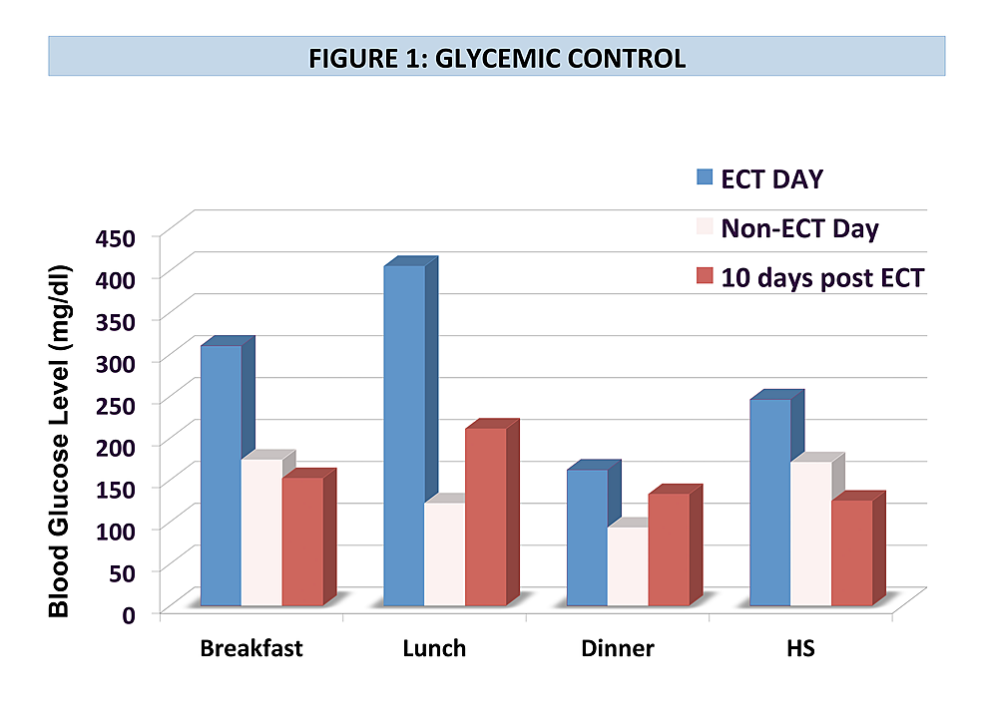
Glycemic Control ECT: Electroconvulsive therapy

**Figure 2:**
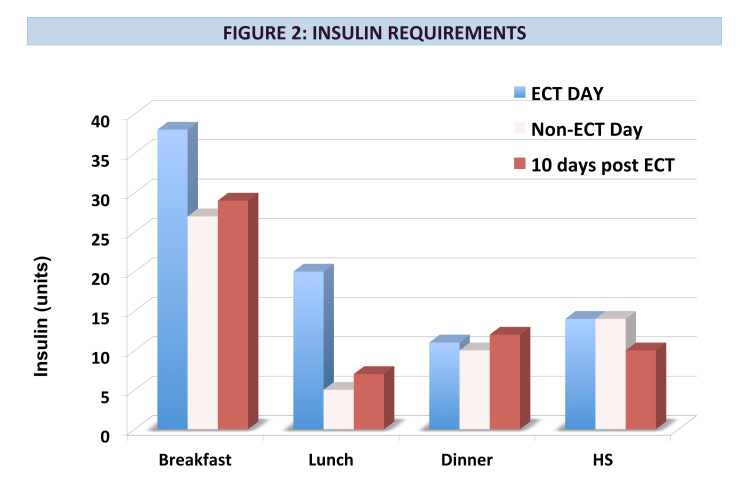
Insulin Requirements ECT: Electroconvulsive therapy

Initially, treatment with glargine 40 units daily and aspart 10 units with meals was started. This was adjusted to glargine 28 units and aspart 12 units with meals when ECT commenced. ECT treatments occurred three times a week for a total of 13 treatments. Significant glycemic variability was noted during hospitalization, with hyper- and hypoglycemia despite consistent carbohydrate intake and stable activity. Near-daily adjustment of insulin doses was required. Pre-dinner hypoglycemia was noted on non-ECT days, prompting distinct “ECT day” and “non-ECT day” insulin regimens. For example, on one ECT day, his mean blood glucose (BG) was 280 mg/dl (range 162-405 mg/dl) and he received 83 units of insulin. The following day (a non-ECT day), his mean blood glucose was 140 mg/dl (range 93-174 mg/dl) with a total daily insulin dose of 56 units (Figures 1-2).

Marked improvement in mood was noted over the course of the hospital stay and the occurrence of delusions abated. Ten days after completion of ECT, insulin requirements were down to 58 units of insulin per day with a mean BG of 155 mg/dl (range 125-211 mg/dl) (Figures 1-2). This represents a decrease in mean BG of 44.6% and a decrease in total daily insulin dose of 32% after the completion of ECT treatment.

## Discussion

The association between diabetes and depression is well-established, with studies demonstrating that these two conditions occur together twice as frequently as would be predicted by chance alone (OR = 2.0, 95% CI 1.8-2.2) [1]. In addition, a meta-analysis by Lustman et al. [4] of 24 studies and over 2800 subjects showed a statistically significant association between depression and degree of hyperglycemia in both type 1 and type 2 diabetes. A meta-analysis of 27 studies with over 5000 patients with type 1 and 2 diabetes by Holt and colleagues showed depression was significantly associated with diabetes complications, including retinopathy, nephropathy, neuropathy, macrovascular complications, and sexual dysfunction [2]. 

While the relationship between depression and diabetes is likely bi-directional, the underlying pathogenesis remains unknown, suggesting a complex interaction of biological, psychological, and genetic factors. Multiple mechanisms have been proposed, including hypothalamic-pituitary-adrenal axis dysfunction, sleep disturbance, inflammation, the stress-induced release of glucocorticoids and catecholamines, environmental and lifestyle factors, and the metabolic effect of antidepressants. However, strong evidence to support any one specific theory does not exist [2]. It has been shown that worsening depression can negatively impact diabetes self-care, leading to poor medication and dietary adherence [5]. This was confirmed by Gonzales and colleagues in a meta-analysis of 47 studies with 17,319 subjects, which showed that depression was associated with significantly reduced diabetes treatment adherence [6]. Prospective, longitudinal studies are needed to clarify the pathways that mediate the association between diabetes and depression. 

The effect that the treatment of depression has on glycemic control in people with diabetes appears to depend on the treatment modality. The combination of psychotherapeutic and psychoeducational interventions has been shown to be cost-effective [7] and yield beneficial mental health outcomes, with improvements in glycemic control [8]. On the other hand, while pharmacotherapy has shown effectiveness in treating depression, there is no clear evidence that it improves glycemic control [3]. There is limited data on the glycemic effects of ECT, which is generally reserved for the treatment of severe depression when medical therapy is ineffective or contraindicated.

Fakhri and colleagues [9] were among the first to observe the favorable effect of ECT on glycemic control in people with diabetes. In 1966, he described a case of a 50-year-old man with a recent diagnosis of diabetes who received two ECT treatments for his depression. Five days after treatment, fasting blood glucose normalized and continued to be normal 14 years later. In a subsequent case series, 8 out of 14 patients had complete remission of hyperglycemic symptoms after one or two ECT treatments [9]. Similarly, Thomas and colleagues reported a 41-year-old with diabetes on insulin, who, four weeks following a course of ECT, was insulin-free with a normal glucose tolerance test (GGT), and remained diabetes-free one year later [10]. 

Other studies have suggested ECT worsens glycemic control, particularly in the short term. Reddy and Nobler [11] presented a case of a 34-year-old without known pre-existing diabetes who developed extreme hyperglycemia after the fourth ECT session, ultimately requiring insulin. Similarly, Yudofski and Rosenthal reported a case of a person with diabetes whose insulin requirements increased dramatically during ECT treatment [12]. 

There are multiple mechanisms by which ECT could affect glucose homeostasis to account for these conflicting clinical reports. ECT has been shown to induce insulin secretion [13]. This would only be expected to affect glycemic control in people with the capacity for endogenous insulin production. This could account for the results of Fakhri et al.’s case series, in which all eight of the ECT responders had non-insulin-dependent DM (NIDDM) or recent-onset DM, while the six non-responders had insulin-dependent DM (IDDM) [9]. It has also been postulated that ECT results in seizure-induced secretion of counter-regulatory hormones that antagonize the effect of insulin, including catecholamines [14-15] and cortisol [16]. The acute increase in cortisol after ECT is well-established and reproducible with repeated treatments, with peak cortisol levels occurring 30 minutes after the induced seizure [17]. This increase in counter-regulatory hormones could account for the development of new or worsening hyperglycemia in people treated with ECT, particularly in the short term. Effective treatment of depression via ECT may also cause favorable changes in glucose metabolism. Depression is associated with insulin resistance and high levels of cortisol and ACTH. Successful treatment of depression could improve or normalize glucose tolerance by reducing the levels of these diabetogenic hormones [18]. Strict dietary modifications and weight loss in the hospital during the course of treatment may also improve glycemic control [19].

Our case is useful in clarifying the immediate and longer-term effects of ECT in a patient without endogenous insulin production. Acutely, ECT increased insulin resistance and requirements, necessitating substantially different insulin doses on ECT days compared to non-ECT days. However, ECT had a positive long-term effect on his glycemic control, with an improvement in mean blood glucose by over 40%. There was also a long-term improvement in insulin resistance, with over 30% reduction in total daily insulin dose. This suggests that effective treatment of depression via ECT may have beneficial effects on improving glycemic control in people with type 1 diabetes.

## Conclusions

Electroconvulsive therapy (ECT) is a major type of treatment for refractory depression and other severe psychiatric diseases. Depression is a common comorbid condition of diabetes. However, the evidence on the effect of ECT on glycemic control in patients with diabetes is limited and conflicting, with reports of both worsening and improvements of hyperglycemia. Our case helps clarify the glycemic effect of ECT in patients with type 1 diabetes. While acutely there was an increase in insulin resistance following an ECT treatment, effective treatment of depression with ECT improved glycemic control and reduced insulin resistance. Given the multiple potential mechanisms by which ECT can alter glucose metabolism, it is important to closely monitor blood glucose during ECT treatment, particularly in patients with diabetes. Treatment of depression may have beneficial effects on improving glycemic control in patients with T1DM.
